# Role of the Drying Technique on the Low-Acyl Gellan Gum Gel Structure: Molecular and Macroscopic Investigations

**DOI:** 10.1007/s11947-018-2210-6

**Published:** 2018-11-19

**Authors:** Mattia Cassanelli, Valentina Prosapio, Ian Norton, Thomas Mills

**Affiliations:** School of Chemical Engineering, University of Birmingham, Edgbaston, Birmingham, B15 2TT UK

**Keywords:** Drying, LA gellan gum, Gel microstructure, Rehydration

## Abstract

The effect of three drying processes (freeze, oven and supercritical CO_2_ drying) on CP Kelco low-acyl gellan gum gel was investigated, highlighting the role of the water removal mechanism (i.e. sublimation, evaporation and solvent replacement/extraction) and the process parameters on the gel structure, rather than focusing on the drying kinetics. It is the first time that a research paper not only compares the drying methods but also discusses and investigates how the molecular and macroscopic levels of gellan gum are affected during drying. Specifically, the dried gel structures were characterised by bulk density and shrinkage analyses as well as scanning electron microscope (SEM) and micro-computed tomography (μCT) microscopy. Micro-differential scanning calorimetry (μDSC) was used in a novel way to investigate the effect of the drying technique on the polymer disorder chains by partial melting of the gel. The resulting water uptake during rehydration was influenced by the obtained dried structure and, therefore, by the employed drying process. It was found that freeze-dried (FD) structures had a fast rehydration rate, while both oven-dried (OD) and supercritical CO_2_-dried (scCO_2_D) structures were slower. After 30 min, FD samples achieved a normalised moisture content (NMC) around 0.83, whereas OD and scCO_2_D samples around 0.33 and 0.19, respectively. In this context, depending on the role of the specific hydrocolloid in food (i.e. gelling agent, thickener, carrier), one particular dried-gel structure could be more appropriate than another.

**Graphical abstract:**
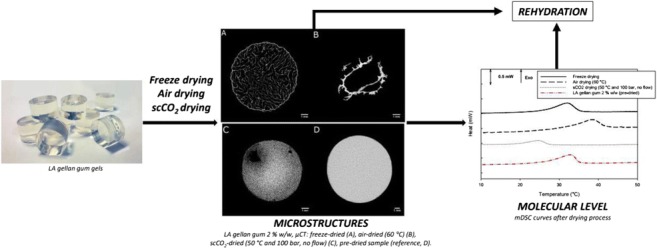
From left to right: unprocessed hydrogels; μ-CT images of dried gels and unprocessed hydrogel; DSC curves after drying process

From left to right: unprocessed hydrogels; μ-CT images of dried gels and unprocessed hydrogel; DSC curves after drying process

## Introduction

Dried foods have been widely produced to extend the product shelf life and to be consumed on demand (Ratti 2001; Marabi et al. 2004). In relatively complex products, such as instant foods or dairy products, the formulation may contain additives and preservatives as ingredients, which need to be considered during the drying process (de Vries 2002; Norton and Foster 2002; Renard et al. 2006). In this context, hydrocolloids are often used in food formulations to modify the product properties, acting as thickener, stabiliser or gelling agents (Phillips and Williams 2004, 2009), and to improve the dried product quality and shelf life (Brown et al. 2010).

Dried foods often need to be rehydrated before consumption (Marabi et al. 2006; Joardder et al. 2015), and the speed of this process can vary according to the specific application. In this light, both the properties and the structure of the dried gel contained in the product formulation may affect the water uptake. Thus, the rehydration rate of both the gel and, therefore, the final product can be modulated, since it is strictly dependent on the drying process (Marabi et al. 2004). Dried gel systems are often used also as carriers for active ingredient release (e.g. drugs, bioactive molecules or sugars) (Hoffman 1987; Nishinari and Fang 2016; Lin and Metters 2006; Tønnesen and Karlsen 2002) and for packaging applications (Suderman et al. 2016).

Dried gel systems are of interest not only in the food industry, but also in biomedicine, and especially in tissue engineering. The design and modulation of biopolymeric dried scaffolds with different patterns and structures may functionalise the material, providing specific mechanical and chemical responsive properties (Sachlos and Czernuszka 2003).

A common gelling agent in the food industry is low-acyl or deacylated (LA) gellan gum. It is a microbial polysaccharide presenting as a primary structure a tetrasaccharide unit composed of glucuronic acid, rhamnose and glucose (Morris et al. 2012). The molecular structure leads to specific mechanical properties that can be further engineered if it is blended to high-acyl (HA) gellan gum, known to be softer and more elastic due to the presence of acyl substituents (Morris et al. 2012; Phillips and Williams 2009). LA gellan gum properties are direct consequence of the double helix formation during gelation and the ion-induced association of the same helices, forming the junction zones (Morris et al. 2012). The resulting network consists of disordered and flexible chains with few highly ordered domains (Chandrasekaran et al. 1988). Reducing the amount of water, the gellan gum chains aggregate and the network becomes more packed (Morris et al. 2012).

Among the industrial drying techniques, freeze drying is widely used in the food industry since, being a non-thermal method, it preserves the food structure (Evans 2009; Krokida et al. 1998) and nutrients (Avila and Silva 1999). The process is based on the freezing of the product, followed by sublimation of the ice crystals into vapour at reduced pressure (Scherer 1990). Throughout the process, the capillary stress is avoided, preventing the collapse of the structure and minimising the shrinkage of the material (Krokida et al. 1998). Another common technique is air drying, which generates a compacted structure (Joardder et al. 2015; Krokida and Maroulis 1997), since local stresses within the material are produced on evaporation (Scherer 1990). This mechanism is affected by the surface tension of the liquid (Deshpande et al. 1992), which is relatively high for water. Supercritical fluid (SCF)-assisted techniques have been proposed recently in the food industry (Brown et al. 2010; Brown et al. 2008), as they are generally used for other food applications, such as extraction of active ingredients from natural resources (Peker et al. 1992), isoelectric precipitation of proteins (Hofland et al. 2000), micronization (Prosapio et al. 2016), and substrate impregnation (De Marco and Reverchon 2017). The general employed fluid is carbon dioxide (CO_2_), being an inert gas, with a relatively low critical point (31.1 °C and 73.8 bar). By changing both pressure and temperature, it is possible to modulate the fluid properties, such as viscosity and density (Wang 2008; Benali and Boumghar 2014; Brunner 1994). For drying applications, the hydrogel-alcogel transition is carried out, followed by solvent removal using supercritical CO_2_ (Ulker and Erkey 2017).

In terms of produced dried-gel gellan gum structure, the aforementioned drying techniques have already been investigated. Tiwari et al. (2015) and Silva-Correia et al. (2011) reported information about freeze-drying in gellan gum systems. However, their analyses were based only on scanning electron microscopy (SEM) observations, lacking data about porosity throughout the whole dried-gel volume. In order to have a 3D sample reconstruction, micro-computed tomography (μCT) can be a useful method to complete the freeze-dried microstructure understanding. Ratti (2001) accurately compared freeze drying with air drying, suggesting that the former is more suitable to achieve a high-quality product although it is more expensive. The effect of air drying on the material structure has been deeply explained and rationalised by Joardder et al. (2015). The drying kinetics and the effect of the different drying processes on the dried-gel macrostructure were reported by Sundaram and Durance (2008). However, in that work, locust bean gum, pectin, and starch were the hydrocolloids investigated and they were mixed to form a single gel system, which is considerably more complex and different from LA gellan gum in terms of gelation mechanism and molecular configuration. Brown et al. (2010) studied the effect of the supercritical CO_2_ (scCO_2_) drying on agar gels in comparison with both oven and freeze drying processes. However, this system was characterised only at the macroscopic scale by μCT, without providing information on the effect on the molecular and network levels. Furthermore, the collected data on agar are significant only for gelling agents with comparable gelation mechanism and molecular structure, unlike gellan gum.

In this work, for the first time, the effect of freeze, oven, and scCO_2_ drying on gel microstructure at both the molecular and macroscopic levels was investigated. Precisely, the role of the physical mechanism of water removal for the specific drying method was highlighted (i.e., sublimation, evaporation, and solvent replacement/extraction), rather than the drying kinetics. LA gellan gum was used as a model gelling agent in a quiescent form, yet other hydrocolloids are expected to behave similarly, especially if they present a similar gelation mechanism, based on the physical interactions of the polymer chains (Gulrez et al. 2011), such as carrageenan (Aguilera and Stanley 1999). This study proposes micro-differential scanning calorimetry (μDSC) analysis as a method to investigate the role of the drying technique on the gel network, characterising the molecular aggregation extent and structure order. In fact, although μDSC has already been used for gellan gum investigations (Sudhamani et al. 2003), it has never proposed for dried gellan gum gels to show the presence of possible changes in the dried sample thermal behaviour. After drying, the samples were rehydrated to study how the structure affects the water uptake into the material.

## Materials and Methods

### Gel Preparation

Low-acyl gellan gum was provided by Kelcogel F, CP Kelco, UK. After heating distilled water to 85 °C, the LA gellan gum powder at 2% weight/weight (*w*/*w*) was slowly added to avoid the formation of clumps. At complete hydration, the solution was poured into sample moulds (13.5 mm in diameter and 65 mm in height) and left to cool at room temperature (20 ± 1 °C). After setting, a maturation period at room temperature (20 ± 1 °C) for 24 h was performed. The gels were cut with a knife in samples of 10 mm in height. All the materials were used with no further treatment or purification.

### Freeze Drying

The gel samples were frozen in a − 18 °C freezer (LEC U50052W, UK) for 24 h, applying a freezing rate of around 0.2 °C/min, previously measured by use of thermocouples (Cole-Parmer® Instrument Company, USA) at both sample core and surface. Afterwards, they were placed onto shelf trays at room temperature in the freeze dryer (SCANVAC 110-4 PRO, LoboGene, UK) for 48 h. The experiments were carried out in a conservative freeze-drying cycle, without providing additional heat from the shelves in order to avoid potential structure collapse. The chamber pressure was set at 0.18 mbar and the temperature of the condenser at − 110 °C, condition that is defined by the equipment. After the drying process, the samples were stored in a desiccator with silica gel beads under low vacuum conditions until characterisation.

### Oven Drying

Oven drying was performed in a vacuum oven (Fistreem International Co. Ltd., Leicestershire, UK) at 20, 40, and 60 °C under static air, room pressure and a constant relative humidity (RH) of 20%. Since oven drying kinetics depends on the temperature, the process time was set accordingly to reach a normalised moisture content value lower than 0.1, as described in the “Moisture Content and Water Activity” section.

### scCO_2_ Drying

Carbon dioxide was supplied from BOC (Guildford, UK). Before drying using scCO_2_, an ethanol (purity 99.9%, AnalaR NORMAPUR, VWR, UK) pre-treatment was performed to replace water, while the supercritical CO_2_ drying was carried out to remove the liquid ethanol from the sample and, therefore, to obtain a solid dried matrix (normalised moisture content (NMC) below 0.1). The gel samples were left, stepwise, in the alcoholic solutions at 25, 50, and 80% wt. Each step was carried out for 6 h, before using absolute ethanol for 24 h. This gradual pre-treatment was needed to reduce the shrinkage extent due to the use of ethanol.

The supercritical drying process necessary to remove ethanol was carried out in two configurations: batch and semi-continuous. In the batch configuration, alcogels, obtained from a high-pressure pipe, were placed into the vessel and then it was pressurised with CO_2_ and heated until the desired operating conditions were achieved. The effects of temperature (40–50 °C) and pressure (85–100 bar) were investigated. In the semi-continuous configuration, alcogels were placed in the same high-pressure vessel, which was pressurised applying a continuous CO_2_ flow (1–2.5 L/min) throughout the experiment using an air-driven liquid pump (MS-71, Haskel, USA). The same conditions of pressure and temperature were tested.

In both configurations, temperature was controlled by a thermostatic water bath, in which the rig is submerged. Pressure was monitored by using a manometer, while the CO_2_ flow rate was read by a digital mass flow meter (RHE08, Rheonik, Germany) and adjusted by opening the metering valve downstream, since steady-state conditions were applied. All the experiments in the batch configuration were carried out for 8 h, followed by a 20-min depressurisation. If the forced flow was applied, a 3-h process was performed.

### Moisture Content and Water Activity

All drying methods were assessed by measuring both the sample moisture content and water activity. Moisture content is expressed as NMC (Brown et al. 2010) through the Eq. 1:

1 $$ \mathrm{NMC}=\frac{\left({M}_{\mathrm{d}}-{M}_{\mathrm{s}}\right)}{\left({M}_{\mathrm{o}}-{M}_{\mathrm{s}}\right)} $$

where *M*_s_ is the sample solid mass, *M*_d_ the sample mass after drying and *M*_o_ the hydrogel mass before drying.

Since the gel concentration is known, and equal to 2% *w*/*w*, it is possible to estimate *M*_s_ from *M*_0_. Brown (2010) suggested a value of NMC < 0.1 as the goal to achieve to have negligible moisture content. Equation 1 was also used to monitor the rehydration process, considering *M*_d_ as a function of the rehydration time. At the NMC < 0.1, water activity was measured by using the Aqualab dew point water activity meter 4te (Labcell LTD, UK). Samples were placed into the test chamber at 25 °C, after being crumbled, to analyse *a*_w_ throughout the sample.

### Microstructure Characterisation

The dried gel microstructure was analysed by X-ray μCT and scanning electron microscope (Philips XL-30 FEG ESEM, Netherlands), in conjunction with the analysis of physical/geometrical properties such as bulk density, shrinkage and shape.

High-resolution micro-computed tomography was performed by using the Skyscan 1172 (Bruker, Belgium). This system allows the visualisation and a complete 3D structure reconstruction of 2D cross-sections without any chemical fixation and sample preparation. The acquisition mode can be set at a maximum current of 96 μA and voltage of 100 kV. After binarisation into black and white images, both qualitative and quantitative analyses were performed by using a CT analyser (1.7.0.0), obtaining porosity information on the whole bulk structure.

ESEM FEG (Philips XL30) was used to collect high-quality images of the dried gel structure. The samples were cut after cooling in liquid nitrogen to highlight both the vertical and horizontal cross-section. The maximum voltage was set up to 10 kV and the magnification up to ×1500.

The absolute (true) density of LA gellan gum was measured by using the AccyPyc II 1340 pycnometer (Micromeritics, USA), using helium as a displacement medium.

The gel shape was visually assessed, whereas the gel shrinkage (Eq. 2) was determined by using the paraffin oil (Sigma-Aldrich, UK) liquid displacement method (Yan et al. 2008; Del Valle et al. 1998). This oil is particularly suitable for this application since it is highly hydrophobic, in contrast with the hydrophilic gel structure.

2 $$ \%\mathrm{Volume}=\frac{\rho_{\mathrm{l}}}{\rho_{\mathrm{l}}}\frac{M^{\prime }-{M}_{\mathrm{d}\ast }}{M^{\prime }-{M}_{\mathrm{o}\ast }}\ 100 $$

where *M*′ is the mass of the chamber filled with oil, *M*_d*_ the mass of oil with the dried sample, and *M*_o*_ the oil mass with the non-treated sample. The experimental oil density *ρ*_l_ was found to be 871 kg m^−3^, while the theoretical value lies in the range of 827 and 890 kg m^−3^.

Once the sample volume was calculated, the bulk density of the sample was measured as the mass was known.

### Gel Rehydration

The water uptake was calculated by measuring the sample weight every 6 min for 30 min. A distilled water bath (100 ml) at room temperature (20 ± 1 °C) was used as a medium (Vergeldt et al. 2014). Rehydrated samples were carefully blotted before weighing to remove surface water.

### Micro DSC

A micro DSC 3 evo (Setaram Instrumentation, France) was used to investigate the thermal transitions. The sample was placed in the “close batch cell” (0.6 ± 0.1 g). The reference cell was filled with an equal mass of distilled water. Two sets of analyses were carried out on LA gellan gum gel before drying, from 5 to 80 °C and from 5 to 55 °C, applying a scan rate of 1 °C/min. The latter temperature range was used to isolate the melting of the disordered chains and avoid the “second” thermal transitions that depict the disruption of the junction zones (Picone and Cunha 2011).

The dried samples were rehydrated for 6 h in 100-mL distilled water to enhance the mobility of the polymer disorder chains and observe the thermal event on cooling. In this case, thermal cycles were applied from 5 to 55 °C.

The μDSC curves were presented as an average of the first cycles in triplicate, while the values of transition temperature, enthalpy and entropy were expressed with plus/minus a single standard deviation.

### Statistical Analysis

All the experiments were performed in triplicate and, for each measurement, six dried gel samples were analysed. Data were analysed by one-way analysis of variance (ANOVA) and Tukey’s multiple comparison tests, using SigmaPlot 12.5 Statistical Software. The level of significance was defined as *p* ≤ 0.05.

## Results and Discussion

### Drying

In order to highlight the effect of the drying process on gel structure, the gel formulation was kept constant throughout the experiments. The gel composition may lead to a different drying kinetics and final microstructure, due to a different crystal distribution in the case of freeze drying (Tiwari et al. 2015), case hardening for air drying (Joardder et al. 2015) and different CO_2_ penetration for SCF-assisted technology. The drying efficacy of the three techniques was evaluated in terms of NMC and water activity (*a*_w_) (Barbosa-Cánovas et al. 2008). At the end of the drying processes (time specified in the “Material and Methods”), NMC was below 0.1 ± 0.01 for all samples. Water activity was 0.28 ± 0.01 for oven-dried samples, 0.25 ± 0.05 for freeze-dried samples and 0.20 ± 0.01 for scCO_2_-dried samples; all the values were considerably lower than the threshold of 0.6, below which bacteria and microorganisms cannot grow and proliferate (Barbosa-Cánovas et al. 2008; Rahman 2009). The difference between the drying processes was negligible, within a standard deviation.

### Dried Microstructure: Effect of the Water Removal Mechanism

**Fig. 1:**
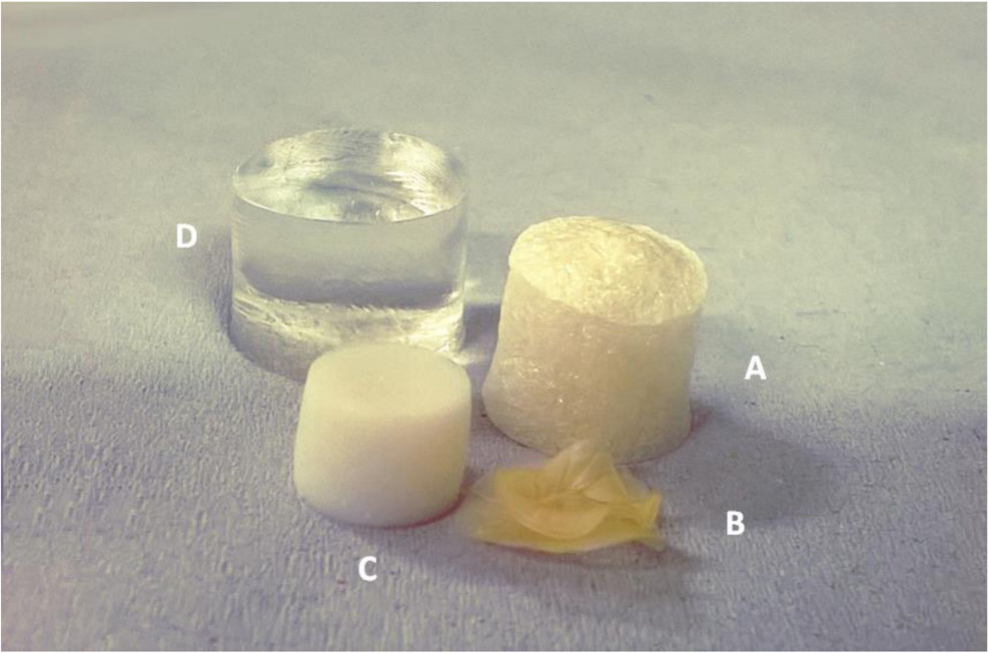
LA gellan gum 2% *w*/*w*, visual comparison: freeze-dried (**a**), oven-dried (60 °C) (**b**), scCO_2_-dried (50 °C and 100 bar, no flow) (**c**), hydrogel before drying (reference, **d**)

The visual appearance of the gels before and after drying is reported in Fig. 1. At a first glance, it is possible to observe that the only drying technique that caused a loss in the cylindrical shape was oven drying, while the other two methods preserved the geometrical features, although some shrinkage occurred. This confirms that water evaporation causes the collapse of the material, while sublimation of ice crystals into vapour and the solvent extraction at supercritical conditions alter the three-dimensional macrostructure less and, therefore, better maintain the cylindrical shape. The absence of capillary stress during the sublimation of ice crystals plays an important role in the microstructure preservation (Scherer 1990) (Fig. 1a). Similarly, this collapse does not occur with supercritical fluid drying due to the absence of the liquid-gas interface (Abbas et al. 2008). On the other hand, during oven drying, the capillary hydrostatic stress due to the surface tension of the receding water menisci causes a collapse of the structure (Fig. 1b) (Snoeck et al. 2014).

**Fig. 2:**
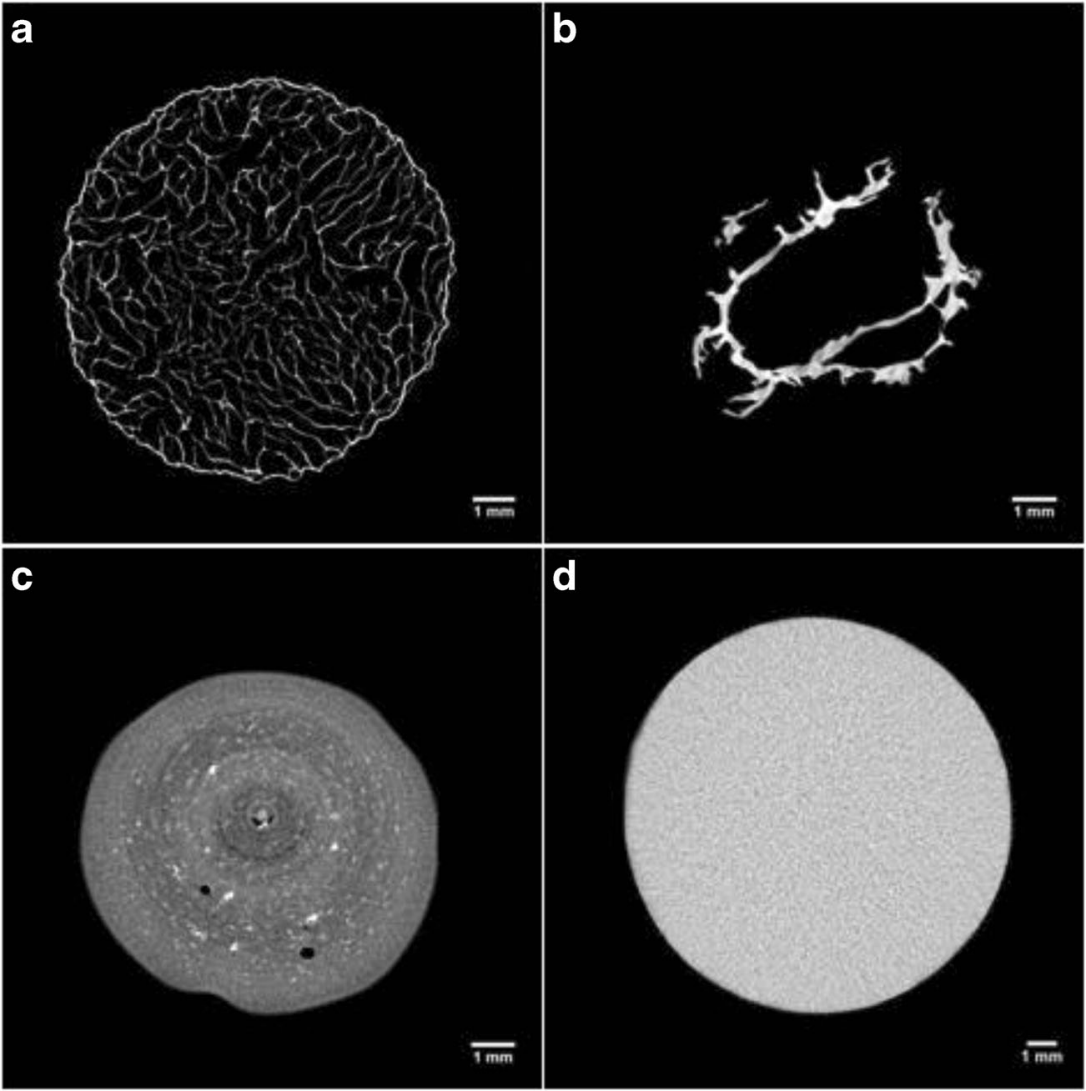
LA gellan gum 2% *w*/*w*, μCT: freeze-dried (**a**), oven-dried (60 °C) (**b**), scCO_2_-dried (50 °C and 100 bar, batch) (**c**), hydrogel before drying (reference, **d**)

The gel microstructure was assessed by μCT to investigate different cross-sections and to obtain quantitative porosity values for the entire bulk volume (Fig. 2a–d). Considerable differences in the dried structure were found among the three drying processes. The μCT analyses revealed that freeze-drying results in a homogeneous structure, with a well-preserved cylindrical shape (Fig. 2a). Oven-dried samples showed an irregular structure formed by thick layers of overlapping material (Fig. 2b). With scCO_2_ drying, the cylindrical shape was retained, although some shrinkage occurred and the internal structure was not completely homogeneous (Fig. 2c). Some larger pores randomly distributed and localised in different regions were observed. This behaviour might be related to the effect of ethanol during the pre-treatment, leading to a stiffer gel structure (Buesa 2008; Eltoum et al. 2001). The use of alcohols can slightly deform the gel, locally modifying the stress generated, especially during the gel shrinkage on drying (Cassanelli et al. 2017). The ethanol pre-treatment is likely to remove all the water from the sample, since the possible presence of liquid water, poorly soluble in supercritical CO_2_, would generate a local collapse due to unbalanced capillary forces in the gel network (Scherer 1990). A heterogeneous structure can be detected with brighter and darker regions in the μCT micrographs. The quantitative μCT analysis highlighted the presence of these larger pores for scCO_2_ drying. Specifically, for freeze drying, the porosity counted 84.8 ± 4.2%, 7.1 ± 0.9% for oven drying and 20.5 ± 8.4% for supercritical carbon dioxide drying; the pore mean size was 480.7 ± 285.6 μm for FD, 2.9 ± 2.1 for OD and 10.4 ± 6.5 μm for scCO_2_D.

**Fig. 3:**
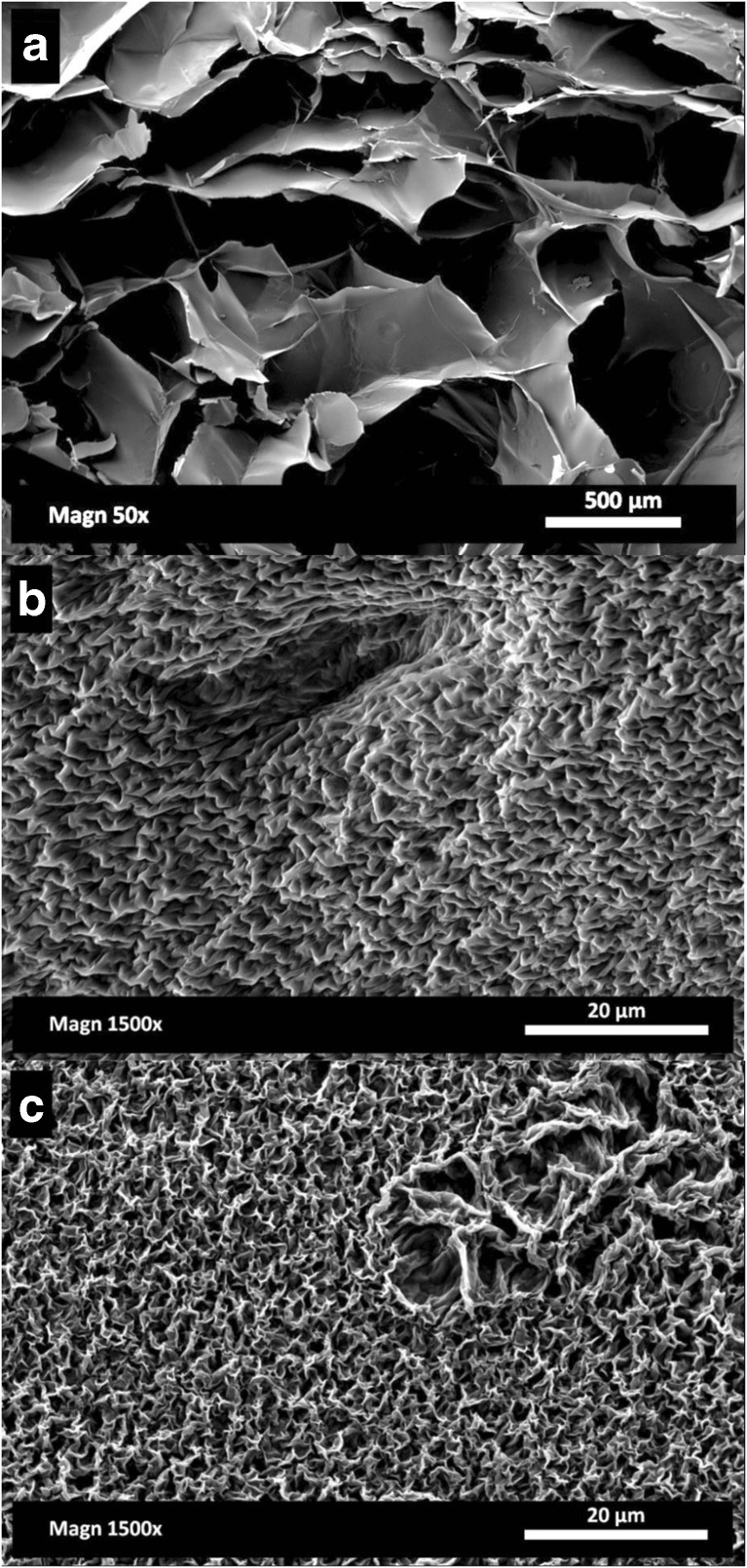
LA gellan gum 2% *w*/*w*, ESEM images: freeze-dried (**a**), oven-dried (60 °C) (**b**), scCO_2_-dried (50 °C and 100 bar, batch) (**c**)

From the ESEM micrographs reported in Fig. 3a–c, it was possible to qualitatively gain information regarding both the pore size and their distribution. In terms of pore size and shape, the freeze-drying results, dependent on the ice crystal size, were in agreement with the current literature on gellan (Silva-Correia et al. 2011). It seems that the internal stresses, generated by evaporation at high temperature during oven drying, induce a structure packing. On the other hand, carbon dioxide in a supercritical state is not expected to lead to a structure collapse (Scherer 1990), despite a homogenous shrinkage, as previously discussed. In this case, the ethanol pre-treatment needs to be considered in the gel shrinkage measurement. In effect, the solvent replacement with ethanol can affect both the shape and volume retention (Buesa 2008; Eltoum et al. 2001). Specifically, before scCO_2_ drying, the direct use of pure ethanol led to more significant shrinkage, up to 50.2 ± 0.6%, while a gradual treatment can limit this effect, reaching a value of 13.1 ± 0.2% (Cassanelli et al. 2017). Obviously, the material shrinkage percentage after scCO_2_ drying needs to take this pre-treatment into consideration, especially if compared with the other techniques (Table 1).

**Table 1:** Bulk densities and shrinkage values

	Freeze drying	Oven drying	Gradual EtOH pre-treatment + scCO_2_ drying (batch)
Bulk density (kg/m^3^)	0 ± 10^a^	17 ± 10^b^	50 ± 10^c^
Shrinkage %	26.20 ± 1.69^a^	82.55 ± 0.12^b^	54.65 ± 1.00^c^

Each value is expressed as mean ± SD (*n* = 3). The values followed by the same superscript letter (a, b, c) in the rows are not significantly different according to one-way ANOVA and Tukey’s multiple comparison tests

The shrinkage extent defines the bulk sample density after drying. The freeze-dried sample had the lowest bulk density, being highly porous. The bulk density for the oven-dried structure was the highest due to its collapse during the drying process. For supercritical CO_2_ drying, the final product was homogeneously shrunk, yet not collapsed. On the other hand, the absolute density was not dependent on the drying process and, in fact, it was found to be 1.7 g/cm^3^ by using the pycnometer, close to the value reported in Upstill et al. (1986).

### Dried Microstructure: Effect of the Process Parameters

In addition to the effect of the water removal mechanism on the gel network, the influence of the process parameters was investigated in terms of the produced dried structure. Considering freeze drying, process parameters such as the pressure chamber and the product temperature could likely affect the drying rate (Chang and Patro 2004), rather than the material structure, which is, by contrast, strongly dependent on the gel formulation. Hence, in this work, all these variables were kept constant. The collapse temperature (Tc) in the freeze drying application is specific for each substance and it is the temperature above which the collapse of the frozen structure occurs. This irreversibly leads to the failure of the material and to defect formation (Kett et al. 2005; Bellows and King 1972; Abdelwahed et al. 2006). During gelation, hydrocolloids generate a network, specific for each gelling agent, in which water is embedded. Specifically, the sol-gel temperature of gellan gum is around 30 °C. As the material temperature in the freeze dryer is expected to be around − 18 °C, the structure stability is likely ensured and its collapse avoided (Figs. 1a and 2a).

**Fig. 4:**
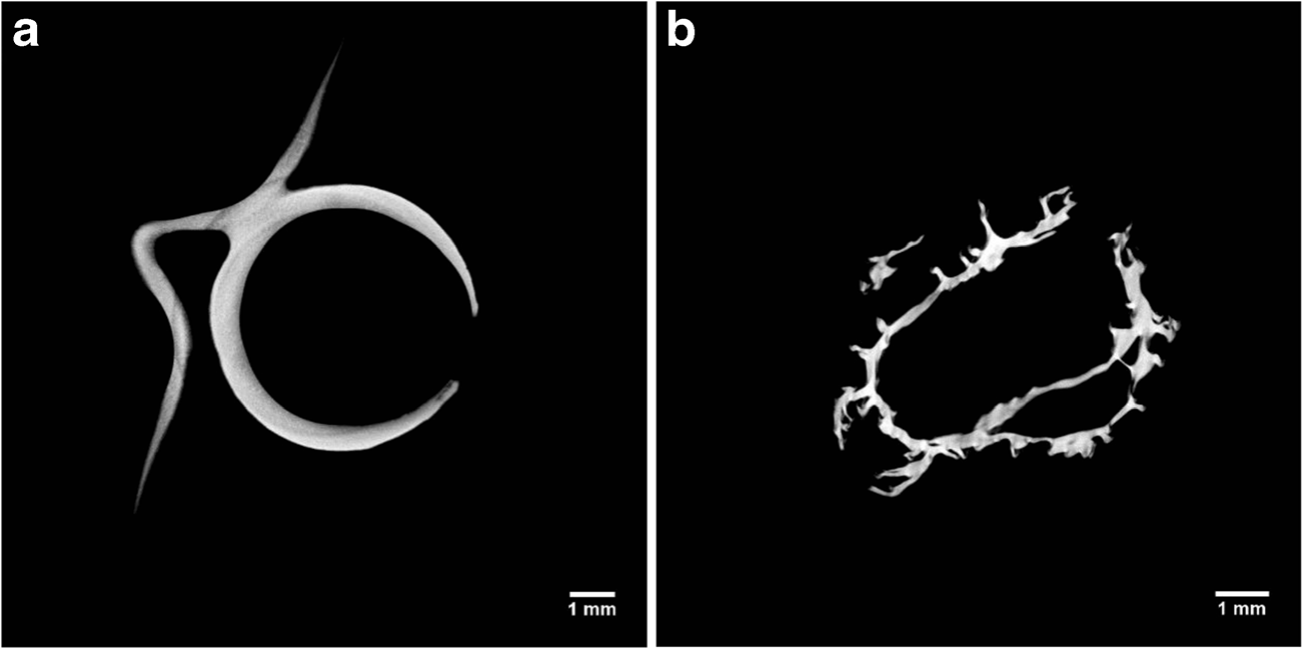
μCT: 2% *w*/*w* LA gellan gum after oven drying at 20 °C (**a**) and 60 °C (**b**)

Oven drying was performed at room pressure and 20, 40, and 60 °C. Similar to freeze drying, it has been reported that both the material formulation and the drying process parameters may contribute to a variation in structure, in terms of porosity, shrinkage and shape retention (Joardder et al. 2015). Focusing on the process variables, temperature should affect the drying rate and consequently the moisture gradient along the material (Joardder et al. 2015). Low temperatures reduce the drying rate and as a consequence the surface evaporation rate is approximately close to the diffusion moisture transfer rate (Mayor and Sereno 2004). On the other hand, higher drying rates may lead to the formation of a rigid crust on the surface, since the moisture content starts to rapidly decrease (Wang and Brennan 1995). It leads to the generation of internal stress, affecting both shrinkage and porosity. This phenomenon is known as case hardening (Joardder et al. 2015). Since oven drying was not vacuum assisted in this work, the main process parameter was temperature, which was set at 20, 40, and 60 °C. The obtained dried microstructure did not show considerable differences in porosity, shrinkage and bulk density within this temperature range (Fig. 4a, b). The total porosity for all these samples was less than 10%. However, the cross-section of the oven-dried structure at 20 °C (Fig. 4a) seemed to have less irregular sample edges. It may be related to a slower water removal (Maskan 2001), inducing less stress and distortion to the material (Joardder et al. 2015).

**Fig. 5:**
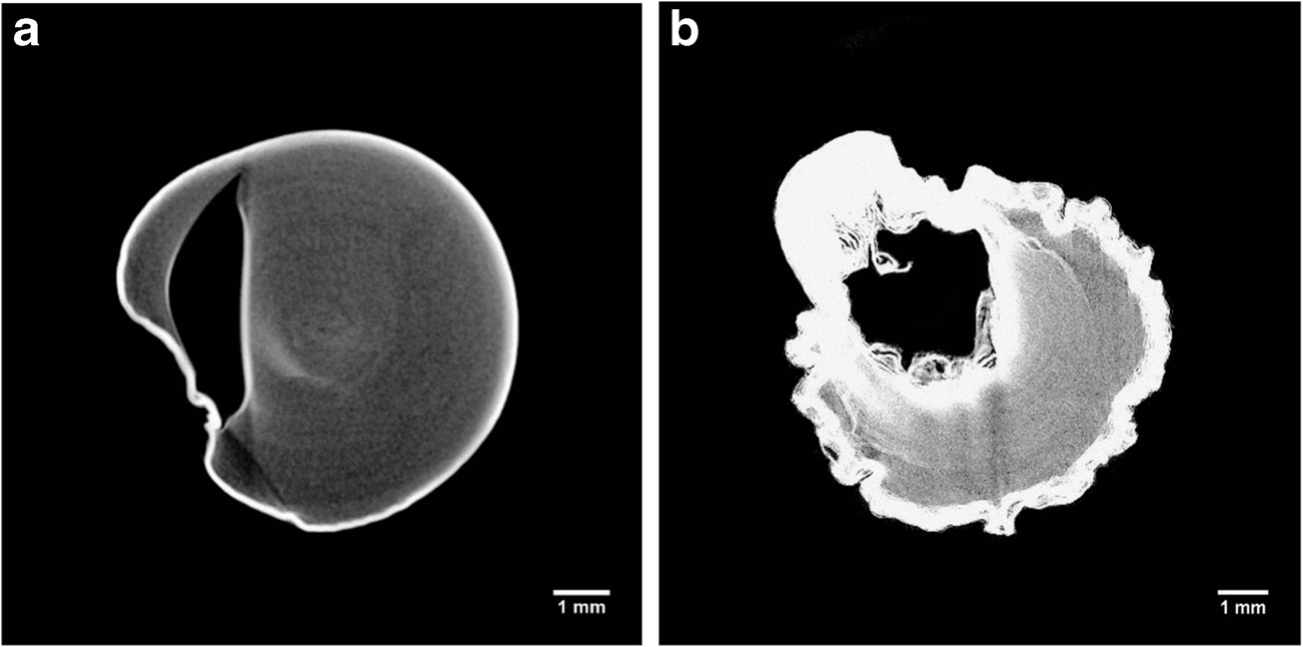
μCT: 2% *w*/*w* LA gellan gum after process in semi-continuous configuration applying 1 mL/min (**a**) and 2.5 L/min flow (**b**). Temperature was set at 50 °C and pressure at 100 bar

The research into supercritical CO_2_ drying has shown that both temperature and pressure may lead to different material structures. However, in this study, the micrographs referred to a process pressure of 85 or 100 bar and a set temperature of 40 or 50 °C did not show structural differences between the samples. It is likely that this temperature/pressure window is too small to generate differences in the dried structure. On the other hand, variations were notable by changing the process configuration, applying a CO_2_ flow rate (Fig. 5a, b). Specifically, from the μCT micrographs, it is possible to observe that the cylindrical section tended to be less defined. Compared to the batch configuration, very large pores were formed. Denser areas were created, since brighter parts were evident, especially on the sample surface. It seems that in some parts of the sample there was a local collapse due to the applied scCO_2_ flow. Furthermore, this flow makes the overall ethanol removal process faster, since after 3 h, the samples were completely dried. The quicker solvent displacement might result in a heterogeneous shrinkage (loss in cylindrical shape) and in a local material densification.

### Gel Rehydration

The water uptake and its diffusion into the material were affected by both the surface and bulk properties (Aguilera and Stanley 1999). The former is more likely influenced by the chemical properties of the gel type and its formulation. On the other hand, the latter considers the mechanical, morphological and physical properties.

**Fig. 6:**
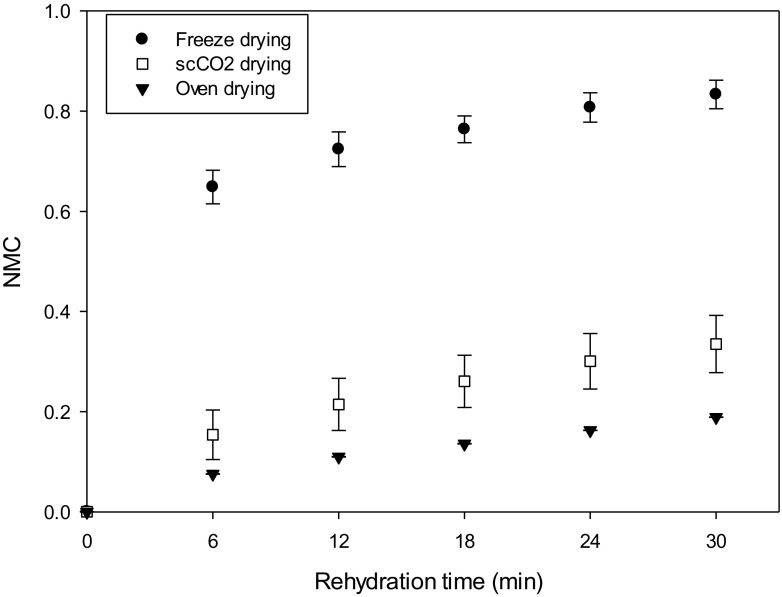
Rehydration curves after different drying processes (black circle) freeze drying, (black down-pointing triangle) oven-dried (60 °C), (white square) scCO_2_-dried (50 °C and 100 bar, batch)

The dried structures were expected to rehydrate differently, based on the porosity distribution, void size and interconnection, shrinkage and consequently bulk density. In Fig. 6, the rehydration curves are shown. Freeze drying resulted in a product that can easily and quickly reabsorb water. This is due to the high porosity and to the presence of large pores, which are a preferential path for water to penetrate. Oven-dried samples rehydrated slowly, since the structure was completely collapsed. No macropores were present and the produced shrinkage was much higher, consequently affecting the water uptake rate. Supercritical carbon dioxide in combination with the ethanol pre-treatment generated an intermediate rehydration rate and water uptake.

Figure 6 suggests that for freeze drying two different rehydration rates, expressed as NMC over time, describe the water uptake. After 6 min, the rate was 0.110 min^−1^, while for longer timescales, the rehydration slowed down to 0.006 min^−1^. This trend became less evident for the other drying techniques. For scCO_2_ drying, this rate passed from 0.15 to 0.04 min^−1^, while for oven drying, it became negligible.

These results suggest that freeze drying is suitable for applications where a fast rehydration is required, while oven-dried and scCO_2_-dried structures are more appropriate for applications where the rehydration rate should be slower.

### Effect on Gellan Gel Molecular Structure

After drying, the structure needs to be rehydrated to enhance the polymer chain mobility and distinguish clear thermal transitions on cooling. Otherwise, flat thermal events would be recorded. For this reason, 6-h rehydrated samples were analysed with μDSC to assess the effect of the drying process on the gel network.

**Fig. 7:**
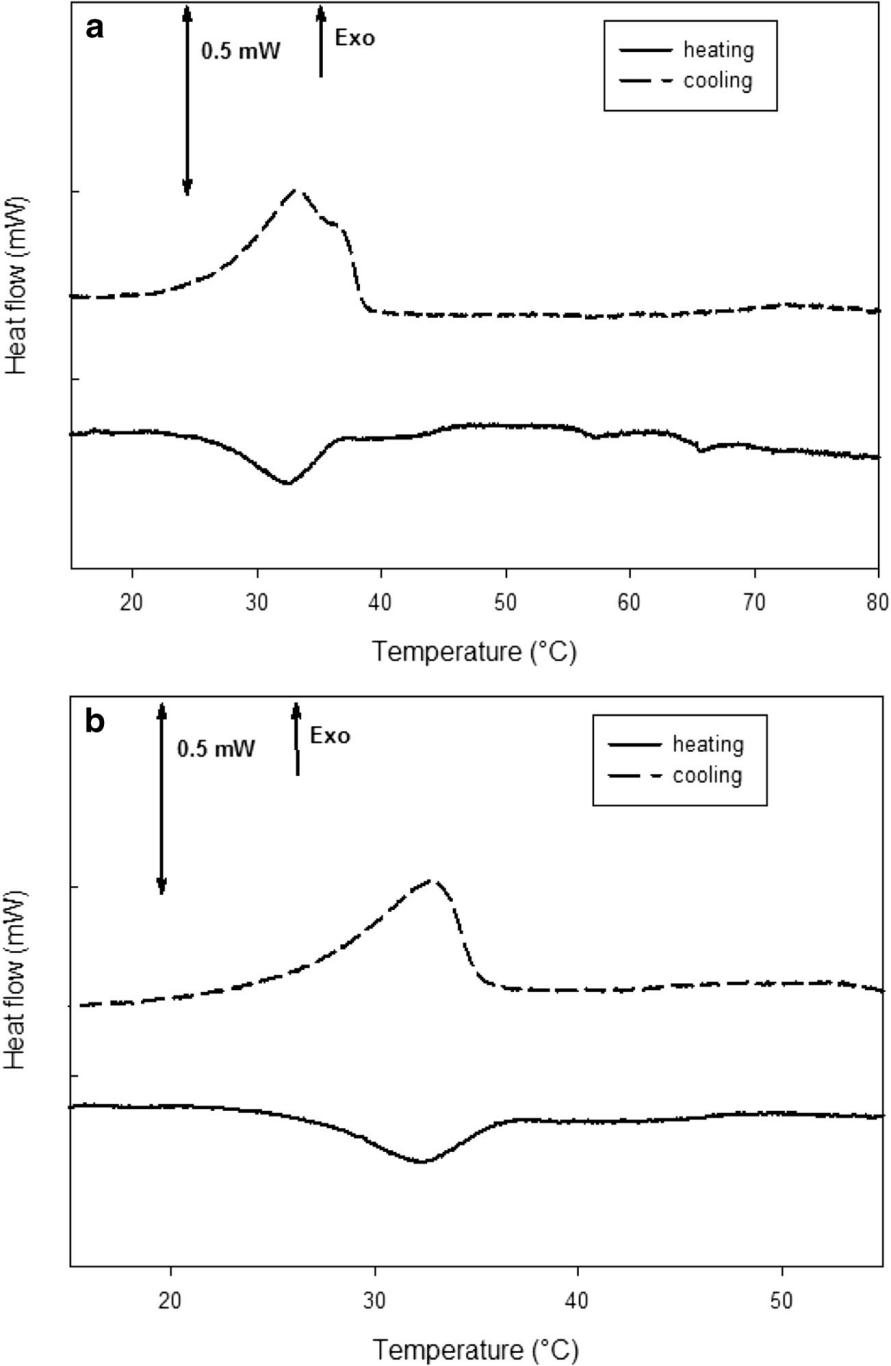
mDSC curves for the gellan gum hydrogel before drying. Heating/cooling cycles were applied from 5 to 80 °C (**a**) and from 5 to 60 °C (**b**)

For the dried gels, the maximum temperature was set at 55 °C with the aim to highlight the effect of the gellan gum disordered domains on cooling (Picone and Cunha 2011). The idea was to induce the helix-coil transition to the disordered chains on heating, without melting the junction zones In effect, if the junction zones were completely melted (Fig. 7a), the enthalpy Δ*H* on cooling was − 0.200 ± 0.005 J g^−1^. Instead, if the gel was heated only up to 55 °C, Δ*H* was − 0.167 ± 0.007 J g^−1^, suggesting that only the disordered chain domains were melted (Fig. 7b). If the gels were completely melted, the shape of the following gelation peak seemed to embed the two transitions (Fig. 7a), namely the coil-helix transition and the chain aggregation in junction zones.

**Fig. 8:**
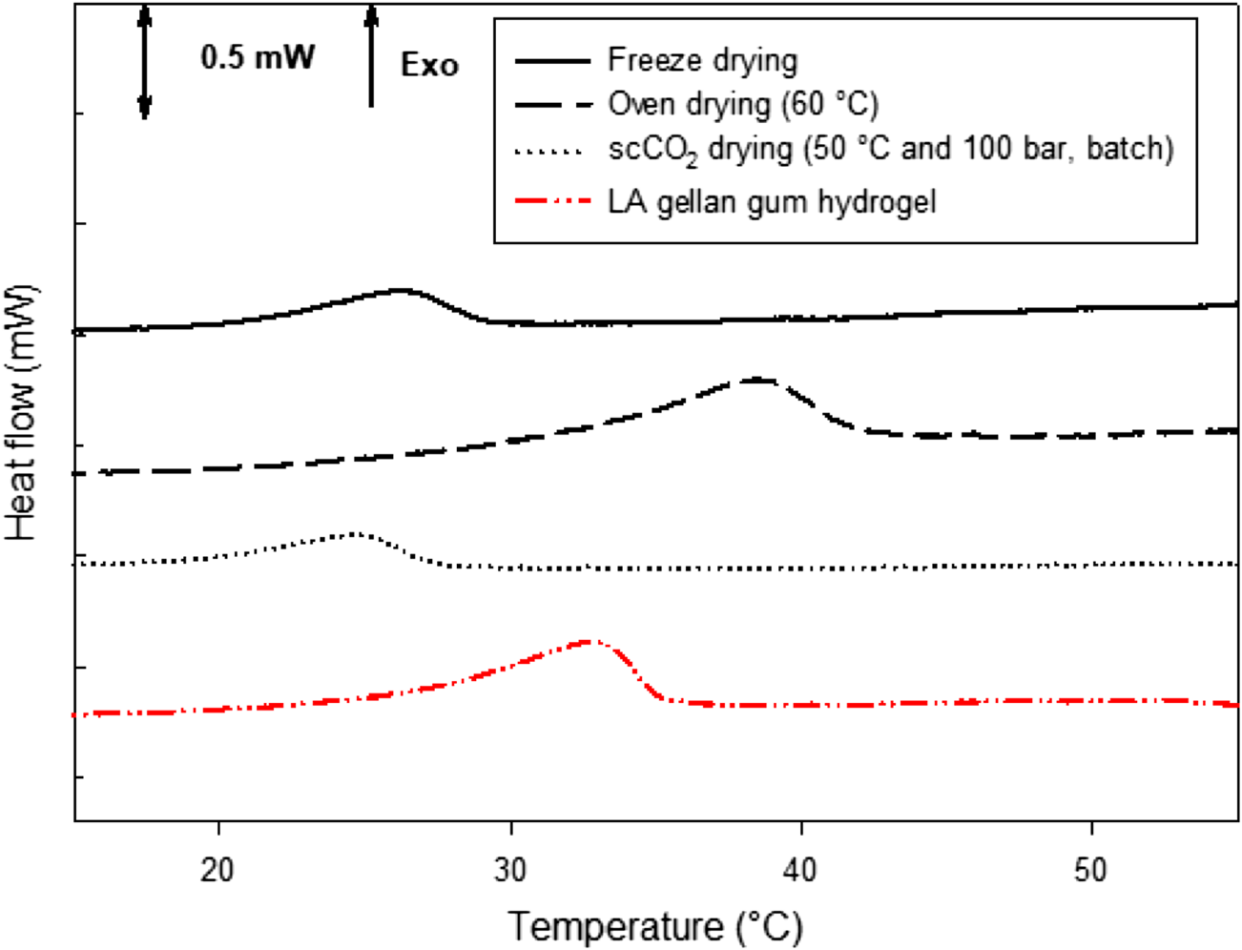
mDSC curves after drying process on cooling

In Fig. 8, micro DSC (mDSC) curves on cooling of samples dried using the different techniques are reported. They suggest that the drying process differently alter the gel structure, since the peak related to the cooling of the disordered chain domains shifted to higher or lower temperatures in comparison to the LA gellan gum hydrogel. It is evident that oven drying sharply increased the thermal transition temperature to 38.9 ± 0.4 °C, compared to the gel before drying (32.7 ± 0.1 °C).

The higher peak temperature compared to the gel before drying is related to the dried gel’s inability to completely reabsorb water because of structure collapse. In effect, the NMC value for these samples was around 0.5 even after 24-h rehydration in distilled water. The oven-dried sample showed that the enthalpy Δ*H*, which is related only to the mobility of the disordered chains if the gel is heated up to 55 °C, as previously discussed, decreased from − 0.167 ± 0.007 to − 0.070 ± 0.017 J g^−1^ (Table 2). It suggests that disordered chains involved in the coil-helix transition are fewer as a result of a more aggregated structure due to the structure collapse and the water removal by evaporation. In terms of entropy difference, Δ*S*, calculated as Δ*H*/*T* at the equilibrium (Deszczynski et al. 2003) on cooling, a reduction was observed, as shown in Table 2. This indicates that, after gel cooling, the system was less ordered, probably due to the relatively high drying temperature, compared to the gel before drying.

**Table 2:** Peak temperatures, enthalpies and entropies for the gel before drying and after the drying process, followed by rehydration for 6 h

	Hydrogel	Freeze-dried	Oven-dried	Gradual EtOH pre-treatment + scCO_2_-dried (batch)
Temperature (°C)	32.6 ± 0.1^a^	27.0 ± 2.5^b^	38.9 ± 0.4^a^	24.1 ± 0.8^b^
Δ*H* (kJ kg^−1^)	− 0.167 ± 0.007^a^	− 0.084 ± 0.029^b^	− 0.070 ± 0.017^b^	− 0.047 ± 0.007^b^
Δ*S* (kJ kg^−1^°C^−1^) × 10^−3^	− 5.1 ± 0.2^a^	− 3.0 ± 0.7^a^	− 1.8 ± 0.4^b^	− 1.9 ± 0.2^b^

Each value is expressed as mean ± SD (*n* = 3). The values followed by the same superscript letter (a, b, c) in the rows are not significantly different according to one-way ANOVA and Tukey’s multiple comparison tests

Freeze drying was expected to force the alignment of the polymer chains during the freezing step along the ice crystal edges, similarly to what occurs during cryogel formation (e.g., xanthan). The reduction in enthalpy to − 0.084 ± 0.029 J g^−1^ compared to the gel before drying suggests that fewer disordered chains are involved in the thermal transition. The peak temperature reduction (27.0 ± 2.5 °C) indicates that the chains should be more aggregated due to the previous formation of ice crystals. However, the overall order of the system slightly decreased, as the entropy was reduced to − 3.0 · 10^−3^ ± 0.7 · 10^−3^ J g^−1^ °C^−1^. This can suggest that the polymer chains in this structure were less ordered although more packed.

Supercritical CO_2_ drying in batch configuration is a gentle process since the capillary stress suppressed. However, the need to perform an ethanol pre-treatment tends to irreversibly influence the polymer conformation, hardening the material (Eltoum et al. 2001; Buesa 2008; Cassanelli et al. 2017). A significant drop to 24.1 ± 0.8 °C in transition temperature was observed, likely due to the presence of ethanol that altered the water network around the polymer, which obstructs polymer rearrangement (Cassanelli et al. 2017). More time was required for the exothermic transition to happen, in comparison with the gel before drying. Similar considerations were applied to the scCO_2_ process in the presence of a continuous flow.

In Table 2, the values of peak temperatures, enthalpies and entropies are summarised. On a second thermal cycle, these dried and rehydrated gels showed a similar thermal behaviour recorded on the first cycle.

## Conclusions and Future Work

The present work shows for the first time the effect of freeze, oven and scCO_2_ drying on low-acyl gellan gum gel systems. All the techniques successfully reduced the water activity below the microbial growth threshold. The drying process influenced the dried gel structure. Specifically, freeze drying generated a highly porous material with more aggregated polymer chains. By contrast, the oven-dried gel was completely collapsed, resulting in a gel that slowly and partially reabsorbs water. The scCO_2_ drying did not induce the structure collapse and only partially shrank the material, leading to a slower water uptake than the freeze-dried gel, yet quicker than the oven-dried gel. However, the necessity to perform an alcoholic pre-treatment made the material harder, changing the polymer network order and increasing the aggregation extent.

The understanding of the relationship between the drying techniques and the produced dried structure can help to design both food products with gelling agent in their formulation and gel agents alone, in either quiescent form or gel particle suspension, throughout the whole production process.

According to with the final application, the most suitable drying technique in terms of produced dried microstructure and the following water uptake might be suggested.

Future works will involve the study of more complex food products, containing hydrocolloids in the formulation. For example, this research can be considered the starting point to design and optimise both the product quality and the production process of freeze-dried ice cream and dairy, dried meet substitutes, ready meals, etc.
